# Nephritis Associated with Scarlet Fever

**Published:** 1908-02-01

**Authors:** 


### SPECIAL ARTICLE.

### NEPHRITIS ASSOCIATED WITH SCARLET FEVER.

1. Whether or not the So-called Febrile Albuminuria is Distinct from Actual Nephritis.

It must always be very difficult to determine whether the albuminuria of fevers is a phenomenon distinct from actual nephritis, or whether such albuminuria is really due to a slight inflammation of the kidney tissues. The patients recover, so that no microscopical examination of the kidneys can be made. When the urines are carefully centrifugalised, an occasional tube-cast is often found, but even then it is a question whether the kidneys would not appear healthy if they could be excised and examined. From a purely academic point of view it seems likely that the trace of albumen found in the urine of many a febrile patient is really due to a slight and transient nephritis, but from a clinical and prognostic point of view there can be little doubt of the value of distinguishing the degrees of albuminuria and of putting the cases into one or other of two groups, namely (1) those with comparatively unimportant albuminuria—practically no nephritis; (2) those with important albuminuria of real significance—actual nephritis.

It is true that the amount of albumen in the urine is hardly a measure of the amount of nephritis, for in some cases there may be severe nephritis without albuminuria at all. These cases are discussed below. Our point at present is that albuminuria alone does not necessarily imply enough nephritis to merit the term from a clinical point of view. These slight cases are characterised by the following features:—

The albuminuria occurs early in the scarlet fever attack—that is to say, during the first few days.

It is transient.

The amount of albumen is small.

There are few tube casts, if any, in the centrifugalised deposit from the urine.

The patient presents none of the other symptoms of acute nephritis.

Stress must be laid, however, upon the fact that it is not possible to exclude actual nephritis merely upon the ground that the albuminuria occurs early. It is generally taught that true scarlatinal nephritis is a complication of convalescence only. This is by no means the case; for, as we shall see presently, acute nephritis, severe enough to prove fatal, may begin during the first week of scarlet fever.

2. The Incidence of Scarlatinal Albuminuria.

It naturally depends upon how frequently the urine is tested in each case of scarlatina whether the proportion of patients found to have albuminuria is high or low. The statistics, therefore, vary greatly. Some observers have gone so far as to test samples of urine three times a day; others have been content with testing one specimen a day; others, one specimen a week. The result is that some have found albuminuria in over 90 per cent. of their scarlet fever cases, others in under 10 per cent. It would appear that, if albumen be tested for once or twice a week in the ordinary way, it will be found in the urines of only 15 out of every 100 cases of scarlet fever. This is important to remember, because the presence or absence of albumen in the urine is sometimes relied on as a point of distinction between scarlet fever, on the one hand, and other erythemata, such as those of rheumatic fever, soap enemata, and the like, on the other. The absence of albumen from the urine upon a single examination is of no value in excluding scarlet fever, though the presence of albuminuria may favour its diagnosis. The above statistics include all forms of albuminuria; let us now consider what proportion of these are clinically cases of nephritis.

3. The Proportion of Scarlatinal Albuminurics in Whom Nephritis is Clinically Present.

Owing to the variability of the statistics upon scarlatinal albuminuria, it is of little value to give the proportion of "simple" to "nephritic" cases. The nephritic cases are almost all detected; the simple cases are detected in larger or smaller numbers according as the urines are examined often or seldom. On the other hand, as will be shown below, it is sometimes extremely difficult to be certain whether nephritis is present or not; cases of slight albuminuria have come to autopsy, and severe inflammation of the kidneys has unexpectedly been found. The following figures are taken from nearly 50,000 consecutive cases in the records of the Metropolitan Asylums Board:—

| | |
|---|---|
| Percentage of total scarlatinal cases in which albuminuria, "simple" or otherwise, was observed ... | 15.1 |
| Percentage of total scarlatinal cases in which the albuminuria was apparently "simple" | 11.2 |
| Percentage of total scarlatinal cases in which the albuminuria was diagnosed as due to nephritis... | 3.9 |

The distinction between "simple" and "nephritic" albuminuria in the above statistics is purely clinical, of course, but apparently when there was the least doubt the case was classed under the heading "nephritis." It seems, therefore, that four out of every 100 cases of scarlet fever are likely to have inflammation of the kidneys; the risk is definite, but luckily it is not great. Nephritis is far from being the commonest complication or sequela of scarlet fever.

4. The Stage of Scarlet Fever at Which Nephritis is Most Likely to Occur.

It is usually taught that acute scarlatinal nephritis is essentially a complication of convalescence, occurring most commonly during the third week after the appearance of the rash. According to the experience of Turner, this is by no means necessarily so; a considerable number of cases, indeed nearly half, occur quite early. In order to avoid the difficulty of deciding between the "simple" and the "nephritic" albuminuria patients, Turner analyses the cases that proved fatal. He finds that, out of 55 patients in whom the existence of nephritis was proved by autopsy,

In 23 the onset of the nephritis was during the acute stage of the scarlet fever.

In 22 the onset was during convalescence.

In 7 the onset was after the fever had subsided one or two days only.

In 3 the date of onset was doubtful.

It appears, therefore, that it is a mistake to suppose that nephritis is a complication of convalescence only. Thus it is most important to examine the urine daily, or every other day, throughout the illness.

5. The Mortality from Scarlatinal Nephritis.

It is difficult to gauge the ultimate mortality from scarlatinal nephritis, for some cases seem to have recovered at the time, and yet ultimately died months or years afterwards, from the effects of renal mischief which may have presented no symptoms meanwhile. Such cases will be mentioned again below. It is more easy to determine the immediate mortality of the complication—that is to say, the mortality within a couple of months. The statistics vary with different years, perhaps owing to variations in the scarlet fever itself. The probability is strongly in favour of the patient's recovery. Out of every 100 cases in whom the diagnosis is clinically acute nephritis, over 80 will get well. The mortality in Turner's cases was 16 per cent. of all the cases of scarlatinal nephritis, or 1 per cent. of all the cases of scarlet fever; that is to say, the chances that a scarlatinal patient will die from acute nephritis arising either during the fever or during convalescence, is about 1 in 100.

6. Influence of Age and Sex Upon Mortality.

If the mortality all round is 1 per cent. the questions at once arise: Is my patient, who is young, more likely to die of scarlatinal nephritis than if he or she were older? Is my patient, who is a female, more likely to die of it than if she were a male? Turner's statistics show clearly that the age has a considerable influence on the prognosis. The older the patient the better it is, provided there be no pre-existent kidney mischief, thus:—

Under 5 years of age the deaths from nephritis were 2.1 per cent. of all the cases of scarlet fever at the same age.
From 5 to 10 years of age 0.9 per cent.
From 10 to 15 years of age 0.5 per cent.
Over 15 years of age 0.2 per cent.

The influence of sex is also fairly well marked, for whilst the numbers of male and female cases of scarlet fever were approximately equal the mortality from nephritis was:—

Amongst males 1.4 per cent. of all the cases of scarlet fever.
Amongst females 0.8 per cent. of all the cases of scarlet fever.

7. Œdema Absent in Most Cases of Acute Scarlatinal Nephritis.

It is very important to know that acute scarlatinal nephritis, even of fatal severity, can occur with practically no œdema at all. In general hospitals the student seldom, if ever, sees a case of acute nephritis without œdema; so prominent is this symptom in most non-scarlatinal cases that the mere mention of acute nephritis brings up the mental picture of a patient with swollen eyelids, lumbar cushion, pitting legs, and œdematous penis and scrotum, or vulva. Most students see but little of the cases in a fever hospital, and in practice afterwards are apt to think the patient cannot possibly have acute nephritis because there is no œdema. Albuminuria in non-œdematous cases, therefore, runs a greater risk than necessary of being regarded as merely febrile, for it is the exception rather than the rule to find general œdema in the cases of scarlatinal nephritis which arise under actual observation in a fever hospital. The same will naturally be the case with patients under treatment in private practice. If nephritis is not diagnosed unless there is œdema to attract attention to it, then there must be cases in which acute scarlatinal nephritis occurs without being diagnosed at all.

This has an important bearing on the occurrence of chronic nephritis in comparatively young people without any apparent cause. Some such cases are no doubt due to the persistence of the after-effects of an acute scarlatinal nephritis that attracted no attention at the time. Turner says: "My own experience is entirely opposed to the view that dropsy is a characteristic symptom. Speaking for the present only of those cases which are treated in hospital from the beginning, I may state that marked œdema never occurs. A slight puffiness of the face is not uncommon, but with fat and chubby children one may be easily deceived, and where present the swelling affects the cheeks, rather than the eyelids, where its presence would be less open to the above doubt. Œdema of other parts—ankles, loins, and scrotum—is absent in the majority of cases." "In one case only can I remember to have seen extensive œdema in a patient who developed nephritis within the hospital. In this case both heart and lungs showed considerable damage before the nephritis developed, and it would be wrong to attribute the œdema to the nephritis alone." It should be mentioned that these remarks apply to the fatal cases, in which the existence of extensive nephritis was proved by autopsy.

It is true that in some cases in which the scarlet fever has been so slight as to escape detection, nephritis, accompanied by considerable œdema, may arise unexpectedly when the patient is up and about, and its scarlatinal origin may be shown by the presence of the characteristic peeling of the skin. The cause of the œdema in these cases may be exertion, chill, or the erect posture, but it seems clear that œdema is usually conspicuous by its absence when the patient is actually under treatment for the scarlatina.

When this is realised one at once sees the importance of having a microscopical examination of the centrifugalised deposit of the urine in every case in which albumen is present at any time during scarlet fever. Casts of the renal tubules, if found in pathological numbers, should indicate the need for treatment as a case of nephritis, such treatment being continued until, at subsequent careful microscopical examinations, renal tube casts are no longer found. Along with the casts it is common to find red corpuscles in the centrifugalised deposit of the urine, even when the blood is not at all obvious to the naked eye.

### 8. Perspiration.

Acute nephritis, as seen in general hospitals, is almost always characterised by a dry skin, from which it is comparatively difficult to evoke sweating. This is otherwise in acute scarlatinal nephritis, at any rate, at the beginning. It is almost the rule to find the onset accompanied by moderate pyrexia, and by perspiration. Later, if the kidney inflammation does not subside, the dry skin may ensue, but this is not the case at the commencement.

### 9. Signs of Bad Prognosis.

Vomiting is not unusual at first, but should it persist, it is one of the gravest symptoms. The amount of urine passed may be much below normal, but much stress must not be laid upon this as a prognostic point, for even in a fatal case the urine may continue pale and abundant. Pallor of the skin occurs within a few days of the start, and is not necessarily a point of bad prognosis. In fatal cases all the symptoms of acute uræmia are to be expected, and it is not at all uncommon to find an erythematous rash all over the body shortly before death. If any *early* symptom of bad prognosis can be picked out from the rest, it is the persistence of vomiting whenever anything is given by the mouth.

### 10. Œdema without Albuminuria.

It is a very true saying that "there are no such things as a 'never' nor an 'always' in medicine." We have just seen that albuminuria without œdema is the rule in scarlatinal nephritis; let us now discuss the exact converse. Quite recently a small child came under observation, the main symptom being general œdema of the subcutaneous tissues from scalp to toes. There was no history of recent scarlet fever in the patient; the child looked like an ordinary case of acute Bright's disease. The urine was obtained by catheter, in the anticipation that it would be found loaded with albumen. To the surprise of everyone, there was not even a trace, and the urine was natural in colour and specific gravity. The diagnosis of nephritis was consequently set aside. The anasarca, however, was due to nephritis after all, as was shown by subsequent examinations of the urine; when the patient had been under observation and treatment for several days, typical desquamation of the skin was noticed, and on several subsequent occasions small amounts of albumen were found on testing the urine, whilst both renal-tube casts and red blood-corpuscles were found in the centrifugalised deposit. The condition was obviously one of acute nephritis following upon a very mild and unsuspected attack of scarlet fever; œdema was a prominent symptom, as we have seen it may be in patients who are up and about and not under treatment for the scarlet fever; had it not been for the œdema, the nephritis would not have been detected at all, and later on the patient might have had chronic nephritis without any assignable cause. The case taught two things at least: First, that even the slightest albuminuria in connection with scarlet fever should at once lead to examination for renal tube casts, seeing that the amount of albumen alone is no measure of the amount of kidney mischief; secondly, that *general œdema in children may be due to acute nephritis, even though an immediate examination of the urine shows that there is no albuminuria.* This is very important, for cases of general œdema in infants without albuminuria are not at all uncommon, and they are frequently regarded as "simple," or at any rate not due to kidney trouble. Many cases are on record. Every year patients of this kind come into hospital. Every practitioner is sure to see such a condition from time to time. Goodhart * has published notes of several cases, in which scarlatina was known to have preceded the œdema by a month or two. In some of his cases no albuminuria was detected on any occasion, and

* Guy's Hospital Reports, 1883.

yet he suggests that the œdema was really nephritic. Other observers hold the same view. The diagnosis is comparatively easy when the œdema without albuminuria is subsequent to recent scarlet fever; it is when the condition occurs after a mild and undetected scarlatina that the diagnosis is more difficult; but when a child is brought in, suffering from general œdema without albuminuria, it must be borne in mind that acute scarlatinal nephritis may occur without any albuminuria at all.

### 11. The Varieties of Post-Scarlatinal Nephritis.

It is usually taught that the typical kidney of post-scarlatinal nephritis is the "large red blood-dripping kidney." This variety, however, is quite rare. More commonly, the organ is, upon the whole, pale, mottled and streaked with dark red; enlarged; with a capsule that peels readily; a smooth surface free from cysts; a cortex which is wider than usual, but with a remarkable lack of definition at the line of junction between cortex and medulla. The appearances at this cortico-medullary junction may be compared to those of the edge of the optic disc in acute optic neuritis—namely, a blurring with much "radial striation." There is no thickening of the arterioles.

Microscopically, several varieties of the disease are described; but to all intents and purposes these are only variations in the degree of one and the same fundamental change. This change is one of *acute interstitial inflammation*, with secondary cloudy swelling and degeneration of the epithelial cells. The acute interstitial trouble may affect the glomeruli alone—a degree of the mischief to which the term "acute glomerular nephritis" is attached; or it may affect not only the glomeruli, but also the interstitial tissue between the tubules both in the cortex and in the medulla. From a clinical point of view there is little or nothing to be gained by trying to distinguish between cases of acute glomerular nephritis and acute interstitial nephritis. The process seems to be identical in each, varying in degree and extent only. When the glomeruli only are affected, they are seen in microscopical sections to be swollen and prominent, the capillary tuft crammed with red corpuscles, with some small-celled exudation betwen the capillaries. When the inflammation is more extensive, the glomeruli are affected as above; but, in addition, there is small round-celled exudation in between the tubules and evenly distributed throughout the organ, with engorgement of the blood-vessels, extravasations of blood both between and within the tubules, cloudy swelling and catarrh of the tubal epithelium, and blockage of many of the tubules with casts composed of blood corpuscles, epithelial cells, and the granular *débris* of cells which have disintegrated. One of the chief points of distinction between this form of acute interstitial nephritis and that variety which is called "acute ascending nephritis" is that in the latter the small-celled infiltration is much more patchily distributed, some portions of the kidney being almost healthy, whilst near by there may be comparatively large collections of small round cells amounting practically to abscesses; whereas in acute scarlatinal nephritis the interstitial change is more uniform.

The above description applies, of course, to the acute stages, and not to the appearances seen if the nephritis has lasted long enough to be termed chronic.

### 12. Bacteriology.

It is well known that certain chemical substances —notably cantharides, turpentine, and oxalic acid —may cause acute nephritis. No such crude chemical bodies can be at work in the scarlatinal cases, but it seems probable that more complicated, and at present unidentified, substances must be the actual cause in these. It is even probable that these unknown substances are of bacterial origin; and it is more than likely that more than one variety of bacteria may produce acute nephritis. It is not extremely uncommon to see acute nephritis associated with acute lobar pneumonia, or with pneumococcal empyema, and from some such cases the pneumococcus has been obtained both from the urine and from the kidney. In some cases of scarlatinal nephritis the streptococcus pyogenes has been cultivated from the kidneys, or even found in smear preparations from them; in many cases no micro-organisms have been detected, but from the fact that if any microbe is found at all it is almost constantly the streptococcus, it would appear likely that this is really the cause of the nephritis. We do not know what is the actual *causa causans* of scarlatina, but we do know that streptococci are at the bottom of many of the lesions associated with it—for example, the tonsillitis; it would not be surprising, therefore, if streptococci are the producers of the toxines which in turn lead to the nephritis.

### 13. The Relation of Nephritis to the Severity of the Scarlatina.

It is by no means only the severest cases of scarlatina that are liable to be accompanied or followed by nephritis. It not infrequently happens that nephritis follows so slight a case that, were it not for this, no notice would be drawn to desquamation, which proves there has been a preceding attack of scarlet fever. This applies rather to the actual scarlatina itself, however, than to its complications; for if there be very severe streptococcal inflammation of the fauces, with glandular infection in the neck, or other streptococcal lesions such as otitis media, pleurisy, and so on, there is an increased liability to streptococcal inflammation of the kidneys. It will often be doubtful whether the nephritis and the other streptococcal inflammations are simultaneous, or whether the nephritis is really secondary to the other sources of streptococci; it is probable that in some cases the nephritis is simultaneous, in others really secondary; but if there be the least evidence of nephritis whilst the patient has any other inflammatory affection such as suppurating glands in the neck, or pleuritic effusion, it is more than likely that streptococci absorbed from these foci will be carried through the body in the blood-stream, and, on reaching the already inflamed kidneys, will increase the damage in them.

14. Surgical Measures.

Under ordinary conditions it is the rule that nephritis is an indication for the surgeon to withhold his hand as far as possible. This is not the case in connection with scarlatinal nephritis, and the rule here should be rather the reverse. If there is any inflammatory condition which surgery can remedy, the presence of acute scarlatinal nephritis is an indication for proceeding with it at once. The cases do exceedingly well as regards the operations themselves, such as the opening of abscesses in the neck or the withdrawal of pleuritic fluid; whilst at the same time the removal of these sources of further infection has a very beneficial effect upon the nephritis. If it is allowed that the nephritis is in all probability streptococcal, at least in many cases, this is exactly what one would expect; for surgical purposes the tissues of a patient who has only recently developed acute nephritis are very different from those of a person who has had deterioration of his arteries and kidneys for years. The existence of scarlatinal nephritis, far from being a contra-indication, is an additional reason for resorting to surgical measures when these can relieve any of the other sequelæ of scarlet fever that may be present.

15. The Occurrence of Chronic Kidney Disease.

This is the last point we will discuss here. The question arises: What will be the ultimate fate of the kidneys of a patient who is now suffering from acute scarlatinal nephritis? As we have seen, the patient may die of the acute attack, though the chances are strongly in favour of recovery. Will the kidneys become perfectly normal again if the acute attack subsides? It is not at all easy to say for certain what proportion of kidneys get perfectly well after they have been acutely inflamed. Clinical evidence is strongly in favour of the view that resolution of the nephritis is as complete as is that of pneumonic lung in the majority of cases. Nevertheless, it is very important to remember that not a few instances of chronic nephritis of young adults seem to be attributable to former scarlet fever. It is quite certain, of course, that chronic nephritis of young adults may occur without any suspicion of preceding scarlet fever; the question is whether scarlet fever is responsible, not for all of the chronic cases, but for any of them. The evidence points to this being the case. The patient may seem to have recovered completely from the acute attack, he may remain apparently well for a number of years, and then develop symptoms of chronic renal mischief, and die. At the autopsy in such a case the kidneys are usually small, red granular kidneys, unless there has been an acute exacerbation of the nephritis before death, when they may be small pale kidneys. Almost every year in the post-mortem room of a large general hospital there is a case of small, red granular kidneys in a patient under 30, or even under 20, years of age; and the history often is that the patient had acute scarlatinal nephritis when young, but was thought to have got quite well. Every now and then these small, red granular kidneys turn up unexpectedly in a patient who has not yet reached puberty, and who came in with symptoms that suggested primary acute Bright's disease; the acute nephritis is proved by the autopsy not to be primary at all, but to have been an acute terminal exacerbation upon the top of a chronic renal affection of years' standing. This difficulty of determining when an acute nephritis is really primary, and when it is an acute exacerbation of chronic mischief that has been latent for years, is a very common problem of clinical medicine. The chronic mischief may have been started by one of many different causes, and amongst the latter is scarlet fever. We have seen how difficult it may be to determine the presence of acute scarlatinal nephritis without repeated urine examinations, owing to the absence of the expected œdema in most cases. The following are two instances in which the origin was known. The first was a boy of 14, who had had scarlet fever as a child, followed by acute nephritis; this had, according to the history, got quite well, and the boy remained in apparently good health until a few days before his death; he was then attacked by vomiting, headache, and drowsiness; coma and convulsions followed, and the boy was admitted to the hospital, obviously moribund from acute uræmia. There was no œdema. The urine contained a small amount of albumen, and a very few tube casts. At the autopsy small, red granular kidneys with numerous small cysts were found, and a hypertrophied left ventricle. In spite of the absence of all symptoms in the interval, there could be little doubt that the kidney cirrhosis had existed for a long time, and in all probability dated from the scarlatinal nephritis which had seemed to get quite well. Microscopically, there was extreme fibrotic change throughout the kidneys. The second case was a woman of 31, who came up complaining of shortness of breath of recent date. She could not account for it herself, and said she had been absolutely well for years. On inquiry, she related that she had had scarlet fever in girlhood, and said that the doctors had told her parents then that "there was something wrong with her water." She died a few weeks later of heart failure, and at the autopsy small red granular kidneys were found, similar both to the naked eye and microscopically to those of the boy above described; her left ventricle was enormous. It seemed certain that the chronic interstitial nephritis was due to the after effects of the scarlatinal nephritis, though the woman had believed herself to be in perfect health during the intervening years.

It is noteworthy that these kidneys are of the small red granular variety. Granular kidney is, in the majority of cases, not the result of a preceding acute nephritis. Acute tubal nephritis is not followed by red granular kidney, but by the small white kidney of chronic tubal nephritis. There is only one form of acute nephritis which is followed by typical chronic interstitial nephritis (red granular kidney), and that is scarlatinal nephritis; this is not surprising when we remember that scarlet fever gives rise, not to acute tubal, but to acute interstitial nephritis.

